# Post‐marketing surveillance of quetiapine fumarate extended‐release tablets in patients with bipolar depression

**DOI:** 10.1002/npr2.12441

**Published:** 2024-04-30

**Authors:** Taro Kishi, Nakao Iwata, Hiroyuki Irie, Masaru Aikawa

**Affiliations:** ^1^ Department of Psychiatry Fujita Health University School of Medicine Toyoake Japan; ^2^ KYOWA Pharmaceutical Industry Co., Ltd. Kita‐ku Japan

**Keywords:** bipolar disorder, delayed‐action preparations, drug‐related adverse reactions, post‐marketing, product surveillance, quetiapine fumarate

## Abstract

**Aim:**

This study aimed to verify the real‐world efficacy and safety of quetiapine fumarate extended‐release tablets (Bipresso® 50 mg and 150 mg; marketing authorization holder is KYOWA Pharmaceutical Industry Co., Ltd., Osaka, Japan) in patients with bipolar depression.

**Methods:**

We performed a post‐marketing surveillance with an observation period of 12 weeks.

**Results:**

In the safety analysis group (*n* = 345), adverse drug reactions (ADRs) occurred in 111 patients (32.17%). The most common ADRs (>1%) were somnolence in 55 patients (15.94%), akathisia in 11 (3.19%), dizziness in 10 (2.90%), weight increase in 6 (1.74%), thirst in 5 (1.45%), and hypersomnia, constipation, and nausea in 4 patients each (1.16%). The only severe ADR was one patient of suicidal ideation, and “longer time since the onset of the first episode” (*p* = 0.011) and “presence of complications” (*p* < 0.001) were identified as significant risk factors for the occurrence of ADRs. In the efficacy analysis group (*n* = 265), the average changes from baseline in the total Montgomery–Åsberg Depression Rating Scale (MADRS) score were −7.3 ± 8.8, −12.2 ± 10.7, −16.8 ± 12.7, and −13.2 ± 12.7 points after 4, 8, and 12 weeks, and at the last evaluation, respectively. The mean MADRS total score decrease had no significant association with maximum daily dose, diagnosis, and presence or absence of prior or concomitant treatment for bipolar disorder with mood stabilizers/antipsychotics/antidepressants.

**Conclusion:**

The efficacy of quetiapine fumarate extended‐release tablets was confirmed in clinical practice, and no new safety concerns or risks were identified.

## Abstract 2

This study assessed the real‐world effectiveness and safety of Bipresso® 50 mg and 150 mg (quetiapine fumarate extended‐release tablets) in bipolar depression patients over a 12‐week observation period. In the safety analysis group (n = 345), 32.17% experienced adverse drug reactions (ADRs), with somnolence being the most common (15.94%). The study identified risk factors for ADRs, including longer time since the first episode and the presence of complications. In the efficacy analysis group (n = 265), patients showed improvements in depression scores over 12 weeks. Quetiapine’s efficacy was confirmed in clinical practice, and no new safety concerns were discovered.

## Graphical abstract

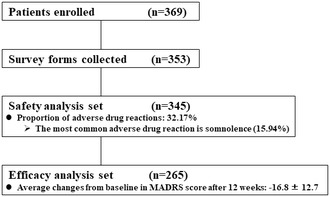

## 1 INTRODUCTION

Bipolar disorder (BD) is an episodic mood alteration defined by episodes of mania/hypomania and depression. The prevalence of this disorder is approximately 0.1–0.4%, and its onset tend to occur late in the second or in the third decade of life.^1^ Moreover, it is likely to relapse, thus the patients must be controlled with appropriate treatment. The diagnosis of a manic or hypomanic episode requires both elevated mood and increased activity. It is essential to confirm the presence of unusually increased activity and energy. Manic episodes are accompanied by marked impairment in social or occupational functioning, possibly requiring hospitalization to avoid harming self or others. In contrast, hypomanic episodes have less prominent impairments. Mania and hypomania are the criteria used to diagnose bipolar I and II disorders, respectively. On the other hand, the accompanying depressive episodes require particular attention, as they may be underdiagnosed or treatment‐resistant and are prone to the risks of the transition to a manic episode or suicide. Even in remission, the patients may exhibit persisting problems of impulsivity and various other alterations of the cognitive function.^1^

Quetiapine fumarate (hereafter, quetiapine) is a dibenzodiazepine‐derivative, synthesized and developed in the United Stated by Zeneca. Quetiapine has an affinity for serotonin 5‐HT_2A_ receptors, dopamine D_2_ receptors, and several other serotonin, dopamine, histamine, and adrenaline receptor subtypes; however, it is characterized by its higher affinity for serotonin 5‐HT_2A_ receptors than for the dopamine D_2_ ones.^2^ In addition, its metabolite, norquetiapine, is a partial 5‐HT_1A_ agonist and a norepinephrine reuptake inhibitor.^3^ Quetiapine is defined as a multi‐acting receptor‐targeted antipsychotic due to its diverse receptor interactions and is a second‐generation antipsychotic. A recent network meta‐analysis demonstrated that quetiapine is effective for the treatment of bipolar mania^4^ and bipolar depression (BDep),^5^, ^6^ and can also be used in the maintenance phase.^7^ In March 2023, the Japanese Society of Mood Disorders (JSMD) released its 2023 Diagnosis and Treatment Guidelines for Bipolar Disorder. This version revised the previous guidelines based on recent domestic and international studies evaluating the efficacy of various drugs for the depressive episodes of bipolar disorder with a high level of evidence and recommend quetiapine as one of the standard drugs.^8^ Moreover, the treatment guidelines published jointly by the Canadian Network for Mood and Anxiety (CANMAT) and the International Society for Bipolar Disorders (ISBD) recommend quetiapine as a first‐line drug for acute bipolar I depression, acute bipolar II depression, and for maintenance in both types.^9^ Additionally, based on the results of our network meta‐analyses,^4^, ^7^ quetiapine has been listed as one of the Essential Medicine by the WHO in 2023.^10^

Although quetiapine was recommended by the domestic and international guidelines as a treatment of depression in patients with bipolar disorder, this indication had not been approved in Japan. Furthermore, a development request was issued by the Ministry of Health, Labour and Welfare (MHLW) in 2010. At that time, quetiapine tablets were approved for the treatment of schizophrenia. However, quetiapine tablets needed to be administered twice or three times a day. Therefore, once‐daily quetiapine extended‐release tablets were selected for development for this indication, as they were expected to result in good medication adherence.

This post‐marketing surveillance was conducted over an observation period of 12 weeks to confirm the real‐world safety and efficacy of the quetiapine extended‐release (Bipresso® extended‐release) tablets, a once‐a‐day sustained‐release matrix formulation to improve patient adherence in individuals with bipolar depression in Japan.

## 2 METHODS

### 2.1 Participants

The study included patients diagnosed, by an enrolling physician, with depressive symptoms of bipolar disorder, with no history of quetiapine use (immediate‐release tablets or the study drug). The enrollment period was from January 2018 to December 2019. The trial period spanned the 2 years and 9 months from January 2018 to September 2020. The study was conducted in accordance with the Good Post‐marketing Study Practice in Japan, using the central registration method and an electronic data collection system.

The target sample size was 300 patients, and the observation period 12 weeks. Previous domestic clinical trials of the study drug (Phase II/III trial CL‐0021, trial of older adults CL‐0022, trial of pharmaceutical formulation switching CL‐0023)^11^ reported various adverse events associated with severe and specific risks (such as “hyperglycemia, diabetic ketoacidosis, or diabetic coma,” “agranulocytosis or leukopenia,” or “hepatic impairment or jaundice”), and a causal relationship to the study drug could not be excluded. These events occurred at a markedly high frequency of approximately 1.0%. Therefore, the sample size was calculated to detect adverse drug reactions occurring at a frequency of 1.0% (with ≥95% reliability) and for which re‐examinations could be collected for a 4‐year period. Moreover, it was assumed that the 300 patients would include at least 100 who were not using the study drug in combination with mood stabilizers.

### 2.2 Study variables

The variables considered were sex, age, body mass index (BMI), diagnosis, and number of episodes in the previous 12 months, time since onset of the first episode, time since onset of the current depressive episode, presence or absence of complications (if present, the diagnosis), presence or absence of other diseases in the medical history (if present, the diagnosis), presence and extent of hepatic or renal impairment, administration status of the study drug (daily dose and frequency, date of first/last dose, changes in the administration route and their reason, termination, or discontinuation), prior pharmacotherapy for bipolar disorder, concomitant drugs, and concomitant therapies. For prior pharmacotherapy of bipolar disorder and concomitant drugs, the total was calculated for the antipsychotic drugs (olanzapine, aripiprazole, and risperidone) and mood stabilizers (lithium carbonate, sodium valproate, carbamazepine, and lamotrigine).

In addition, the clinical test values included as variables were the complete blood count (white and red blood‐cell count, hemoglobin, hematocrit, platelet count); chemistry panels, including total proteins, albumin, total bilirubin, alkaline phosphatase, liver function (alanine transaminase [ALT], aspartate transaminase [AST], and gamma‐glutamyltransferase [GGT]), lactate dehydrogenase, prothrombin time, creatinine kinase, electrolytes (Na, K, Cl), serum creatinine, blood urea nitrogen, urea, serum lipids (total cholesterol, high‐ and low‐density lipoproteins [HDL and LDL, respectively], and triglycerides), prolactin, glucose, and glycated hemoglobin (HbA1c); urinalysis, 12‐lead electrocardiogram, and body weight.

### 2.3 Efficacy and safety evaluation

#### 2.3.1 Safety

The presence or absence of adverse events (including adverse drug reactions and altered clinical or other test results) was monitored for 12 weeks during administration of the test drug and afterwards; we recorded the type of the adverse event, date of occurrence, severity with details, study drug treatment, symptomatic treatment, outcome and its date, causal relationship to the study drug, other possible causes, and test data related to the adverse event, beginning at the start of administration of the study drug. Among the adverse events, we investigated those with a possible causal relationship to the study drug (hereafter, adverse drug reactions [ADRs]). The ADRs were described using the basic terminology and system organ classes found in the Japanese version (MedDRA/J) of the International Council for Harmonisation of Technical Requirements for Pharmaceuticals for Human Use (ICH) medical terminology, version 23.1.

#### 2.3.2 Efficacy

The score of the MADRS was calculated at baseline, and changes from the baseline were evaluated at 4, 8, and 12 weeks, and at last evaluation. The response proportion was defined as the ratio of patients with a ≥50% reduction from baseline in the MADRS total score, and the remission proportion as the ratio of patients with a MADRS total score ≤12 points.

### 2.4 Statistical analysis

The MADRS score summary statistics are presented as mean ± standard deviation (SD). The Fisher's exact test was used for 2 × 2 contingency tables. For 2 × n contingency tables, the chi‐squared test (*χ* ^2^ test) was used for non‐ordinal scales, and the Cochran‐Armitage test for ordinal scales. However, statistical testing was not performed for groups smaller than 10 patients. The two‐tailed significance level for statistical tests was set to 5%. All statistical analyses were performed using SAS software, Version 9.4 of the SAS System for Microsoft Windows (Copyright © 2013–2023 SAS Institute Inc.; SAS and all other SAS Institute Inc. product or service names are registered trademarks or trademarks of SAS Institute Inc).

## 3 RESULTS

### 3.1 Patients

**FIGURE 1:**
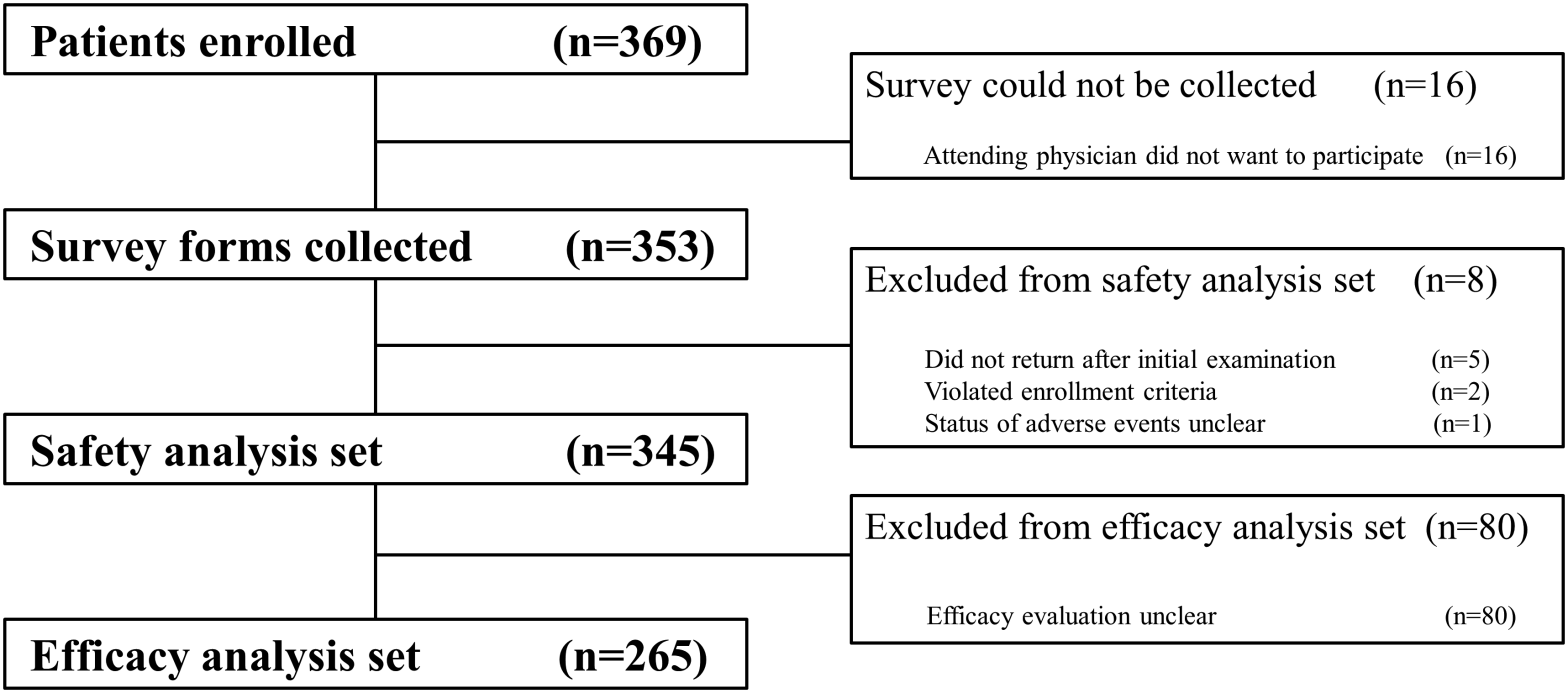
Patient composition. The study involved patients diagnosed by enrolling physicians with depressive symptoms of bipolar disorder and no prior history of quetiapine use. This chart provides numbers regarding enrollment, survey forms collected, and analysis of safety analysis set and efficacy analysis set.

In total, 369 patients were enrolled by December 31, 2019, and among them, survey forms were obtained for 353 patients from 60 facilities; for 16 patients, the survey forms could not be collected because the attending physician declined to participate. Among these 353 patients, eight were excluded: five were lost to follow‐up after the initial examination, two did not meet the inclusion criteria, and one had unclear presence or absence of adverse events; therefore, the safety analysis was conducted on a group of 345 patients. Furthermore, 80 patients were excluded for unclear efficacy, leaving an efficacy analysis sample of 265 patients (Figure 1). Notably, ADRs were observed in two of the eight patients excluded from the safety analysis (one patient of malaise and one of somnolence, both mild).

### 3.2 Patient characteristics

The 345 patients in the safety analysis group comprised 128 men (37.1%) and 217 women (62.9%). The mean age was 42.6 ± 15.0 years (range: 16–85 years), and 31 (9.0%) were ≥ 65 years old.

One‐hundred patients (29.0%) had bipolar I disorder, and 245 patients (71.0%) had bipolar II disorder. The mean number of episodes within the previous 12 months was 1.5 ± 1.1 (range: 0–8), and the mean time since the onset of the first episode was 128.6 ± 109.7 months (range: 2–624 months). Prior pharmacotherapy for the treatment of bipolar disorder was administered to 197 patients (57.1%). The most common prior pharmacotherapies were lithium carbonate (*n* = 74, 21.4%), sodium valproate (*n* = 71, 20.6%), and aripiprazole (*n* = 39, 11.3%).

Overall, 140 patients (40.6%) had complications; the most common (≥1%) were insomnia (*n* = 39, 11.3%), constipation and hyperlipidemia (*n* = 17, 4.9%), hypertension (*n* = 15, 4.3%), headaches (*n* = 10, 2.9%), attention‐deficit/hyperactivity disorder (*n* = 9, 2.6%), chronic gastritis and Parkinsonism (*n* = 8, 2.3%), and fatty liver and dyslipidemia (*n* = 6, 1.7%). Back pain, dysmenorrhea, liver dysfunction, hyperuricemia, iron‐deficiency anemia, migraine, and autism spectrum disorder were noted less frequently (each *n* = 5, 1.4%), followed by alcoholism, gastroesophageal reflux disease, and obsessive‐compulsive disorder (each *n* = 4, 1.2%). Moreover, eight patients had hepatic impairment (2.3%; *n* = 7 mild, *n* = 1 moderate), and eight patients had renal impairment (2.3%, all mild) at baseline.

### 3.3 Status of study drug administration and concomitant drugs or therapies

The status of the study drug administration among the 345 patients in the safety analysis group is shown in Table 1.

**TABLE 1:** Administration status of the study drug.

Variable	Safety analysis set (*n* = 345)	Number of patients	(%)
Initial daily dose	50 mg	309	89.6
	100 mg	7	2.0
	150 mg	19	5.5
	200 mg	1	0.3
	250 mg	0	0.0
	300 mg	9	2.6
	>300 mg	0	0.0
Increase to 150 mg/day	No	181	52.5
	Yes	164	47.5
Increase to 300 mg/day	No	256	74.2
	Yes	89	25.8
Maximum daily dose	50 mg	131	38.0
	100 mg	20	5.8
	150 mg	86	24.9
	200 mg	5	1.4
	250 mg	1	0.3
	300 mg	102	29.6
	>300 mg	0	0.0
Total duration of administration	1–7 days	29	8.4
	8–14 days	29	8.4
	15–28 days	34	9.9
	29–56 days	36	10.4
	57–84 days	18	5.2
	≥85 days	199	57.7

The initial dosing frequency was once daily for all patients; 89.6% had a dosage of 50 mg and 10.4% had 100–300 mg. However, 47.5% of patients had their dose increased to 150 mg/day. The maximum daily dose was 50 mg in 131 patients (38.0%), 300 mg in 102 (29.6%), and 150 mg in 86 (24.9%). The total duration of the treatment was most commonly ≥85 days (*n* = 199, 57.7%), followed by 29–56 days (*n* = 36, 10.4%); 146 patients (42.3%) discontinued use during the 12‐week observation period. The most frequent reasons for discontinuation were the occurrence of adverse events in 76 patients, patient's request (excluding occurrence of adverse events) in 32 patients, and failure to present on the examination dates during the survey period in 23 patients.

Concomitant drugs for bipolar disorder were used by 202 patients (58.6%); the most common were lithium carbonate (*n* = 79), sodium valproate (*n* = 76), and aripiprazole (*n* = 39). Other therapies for bipolar disorder were used in 57 patients, including psychoeducation (*n* = 28), cognitive behavioral therapy (*n* = 7), and interpersonal and social rhythm therapy (*n* = 3).

### 3.4 Safety

#### 3.4.1 Occurrence of adverse drug reactions

The ADRs occurring in each period and for each event are shown in Table 2 (days 1–7, 8–14, 15–28, 29–56, 57–84, and 85+). ADRs were observed in 111 (32.17%) of the 345 patients in the safety analysis group; the most common ones (≥1%) were somnolence in 55 patients (15.94%), akathisia in 11 (3.19%), dizziness in 10 (2.90%), weight increase in 6 (1.74%), thirst in 5 (1.45%), and hypersomnia, constipation, and nausea in 4 patients each (1.16%). Furthermore, a severe ADR of suicidal ideation was observed in 1 patient (0.29%).

**TABLE 2:** Onset time of adverse drug reactions.

	Status in post marketing surveillance
Number of patients in the safety analysis set	345
Number of cases of occurrence of adverse drug reaction	111
Proportion of occurrence of adverse drug reaction	32.17%

Class of adverse drug reaction	Period of occurrence						Total (%)
	1–7 days	8–14 days	15–28 days	29–56 days	57–84 days	≥85 days	
	Number of patients with occurrence by class of adverse drug reaction (%)						
Metabolism and nutrition disorders	0 (0.00)	0 (0.00)	3 (100.00)	0 (0.00)	0 (0.00)	0 (0.00)	3 (0.87)
Hyperglycemia	0 (0.00)	0 (0.00)	1 (100.00)	0 (0.00)	0 (0.00)	0 (0.00)	1 (0.29)
Increased appetite	0 (0.00)	0 (0.00)	1 (100.00)	0 (0.00)	0 (0.00)	0 (0.00)	1 (0.29)
Hyperlipidemia	0 (0.00)	0 (0.00)	1 (100.00)	0 (0.00)	0 (0.00)	0 (0.00)	1 (0.29)
Psychiatric disorders	2 (33.33)	2 (33.33)	0 (0.00)	2 (33.33)	0 (0.00)	0 (0.00)	6 (1.74)
Hallucination visual	1 (100.00)	0 (0.00)	0 (0.00)	0 (0.00)	0 (0.00)	0 (0.00)	1 (0.29)
Initial insomnia	0 (0.00)	1 (50.00)	0 (0.00)	1 (50.00)	0 (0.00)	0 (0.00)	2 (0.58)
Listless	0 (0.00)	0 (0.00)	0 (0.00)	1 (100.00)	0 (0.00)	0 (0.00)	1 (0.29)
Suicidal ideation	0 (0.00)	1 (100.00)	0 (0.00)	0 (0.00)	0 (0.00)	0 (0.00)	1 (0.29)
Bipolar disorder§	1 (100.00)	0 (0.00)	0 (0.00)	0 (0.00)	0 (0.00)	0 (0.00)	1 (0.29)
Nervous system disorders	36 (45.57)	18 (22.78)	15 (18.99)	7 (8.86)	4 (5.06)	1 (1.27)	79 (22.90)
Akathisia	3 (27.27)	3 (27.27)	2 (18.18)	2 (18.18)	1 (9.09)	0 (0.00)	11 (3.19)
Dizziness	5 (50.00)	4 (40.00)	1 (10.00)	0 (0.00)	0 (0.00)	0 (0.00)	10 (2.90)
Dizziness postural	0 (0.00)	0 (0.00)	0 (0.00)	1 (100.00)	0 (0.00)	0 (0.00)	1 (0.29)
Headache	1 (100.00)	0 (0.00)	0 (0.00)	0 (0.00)	0 (0.00)	0 (0.00)	1 (0.29)
Hypersomnia	3 (75.00)	0 (0.00)	1 (25.00)	0 (0.00)	0 (0.00)	0 (0.00)	4 (1.16)
Somnolence	25 (45.45)	11 (20.00)	11 (20.00)	4 (7.27)	3 (5.45)	1 (1.82)	55 (15.94)
Tremor	0 (0.00)	1 (100.00)	0 (0.00)	0 (0.00)	0 (0.00)	0 (0.00)	1 (0.29)
Cardiac disorders	0 (0.00)	0 (0.00)	1 (100.00)	0 (0.00)	0 (0.00)	0 (0.00)	1 (0.29)
Tachycardia	0 (0.00)	0 (0.00)	1 (100.00)	0 (0.00)	0 (0.00)	0 (0.00)	1 (0.29)
Respiratory, thoracic, and mediastinal disorders	1 (100.00)	0 (0.00)	0 (0.00)	0 (0.00)	0 (0.00)	0 (0.00)	1 (0.29)
Dry throat	1 (100.00)	0 (0.00)	0 (0.00)	0 (0.00)	0 (0.00)	0 (0.00)	1 (0.29)
Gastrointestinal disorders	6 (50.00)	2 (16.67)	1 (8.33)	3 (25.00)	0 (0.00)	0 (0.00)	12 (3.48)
Abdominal discomfort	1 (100.00)	0 (0.00)	0 (0.00)	0 (0.00)	0 (0.00)	0 (0.00)	1 (0.29)
Constipation	2 (50.00)	0 (0.00)	1 (25.00)	1 (25.00)0	(0.00)	0 (0.00)	4 (1.16)
Diarrhea	1 (100.00)	0 (0.00)	0 (0.00)	0 (0.00)	0 (0.00)	0 (0.00)	1 (0.29)
Dyspepsia	0 (0.00)	0 (0.00)	0 (0.00)	1 (100.00)	0 (0.00)	0 (0.00)	1 (0.29)
Nausea	2 (50.00)	1 (25.00)	0 (0.00)	1 (25.00)	0 (0.00)	0 (0.00)	4 (1.16)
Tongue dry	0 (0.00)	1 (100.00)	0 (0.00)	0 (0.00)	0 (0.00)	0 (0.00)	1 (0.29)
Hepatobiliary disorders	0 (0.00)	1 (50.00)	1 (50.00)	0 (0.00)	0 (0.00)	0 (0.00)	2 (0.58)
Hepatic function abnormal	0 (0.00)	1 (50.00)	1 (50.00)	0 (0.00)	0 (0.00)	0 (0.00)	2 (0.58)
Skin and subcutaneous tissue disorders	1 (33.33)	0 (0.00)	1 (33.33)	0 (0.00)	1 (33.33)	0 (0.00)	3 (0.87)
Drug eruption	0 (0.00)	0 (0.00)	0 (0.00)	0 (0.00)	1 (100.00)	0 (0.00)	1 (0.29)
Pruritus	0 (0.00)	0 (0.00)	1 (100.00)	0 (0.00)	0 (0.00)	0 (0.00)	1 (0.29)
Rash	1 (100.00)	0 (0.00)	0 (0.00)	0 (0.00)	0 (0.00)	0 (0.00)	1 (0.29)
General disorders and administration site conditions	8 (66.67)	0 (0.00)	2 (16.67)	2 (16.67)	0 (0.00)	1 (8.33)	12 (3.48)
Face edema	1 (100.00)	0 (0.00)	0 (0.00)	0 (0.00)	0 (0.00)	0 (0.00)	1 (0.29)
Feeling abnormal	0 (0.00)	0 (0.00)	0 (0.00)	1 (50.00)	0 (0.00)	1 (50.00)	2 (0.58)
Malaise	2 (66.67)	0 (0.00)	1 (33.33)	0 (0.00)	0 (0.00)	0 (0.00)	3 (0.87)
Edema	0 (0.00)	0 (0.00)	0 (0.00)	1 (100.00)	0 (0.00)	0 (0.00)	1 (0.29)
Edema peripheral	1 (100.00)	0 (0.00)	0 (0.00)	0 (0.00)	0 (0.00)	0 (0.00)	1 (0.29)
Thirst	4 (80.00)	0 (0.00)	1 (20.00)	0 (0.00)	0 (0.00)	0 (0.00)	5 (1.45)
Investigations	1 (12.50)	0 (0.00)	2 (25.00)	3 (37.50)	2 (25.00)	0 (0.00)	8 (2.32)
Alanine aminotransferase increased	0 (0.00)	0 (0.00)	0 (0.00)	1 (100.00)	0 (0.00)	0 (0.00)	1 (0.29)
Aspartate aminotransferase increased	0 (0.00)	0 (0.00)	0 (0.00)	1 (100.00)	0 (0.00)	0 (0.00)	1 (0.29)
Blood pressure decreased	0 (0.00)	0 (0.00)	1 (100.00)	0 (0.00)	0 (0.00)	0 (0.00)	1 (0.29)
Weight increased	1 (16.67)	0 (0.00)	1 (16.67)	2 (33.33)	2 (33.33)	0 (0.00)	6 (1.74)
Injury, poisoning and procedural complications	0 (0.00)	1 (100.00)	0 (0.00)	0 (0.00)	0 (0.00)	0 (0.00)	1 (0.29)
Sedation complication	0 (0.00)	1 (100.00)	0 (0.00)	0 (0.00)	0 (0.00)	0 (0.00)	1 (0.29)
Social circumstances	0 (0.00)	0 (0.00)	0 (0.00)	0 (0.00)	1 (100.00)	0 (0.00)	1 (0.29)
Gambling	0 (0.00)	0 (0.00)	0 (0.00)	0 (0.00)	1 (100.00)	0 (0.00)	1 (0.29)
						MedDRA/J Version (23.1)	

*Note*: If the same patient with the same System Organ Class (SOC) experienced different ADRs (Preferred Terms [PT]) that occurred during the same onset time period, the SOC was counted as one case. If the same patient with the same SOC experienced a different PT that occurred during a different onset time period, the SOC was counted as one case for each onset time period, whereas the total SOC was counted as one case without counting duplicates.

§: exacerbation of bipolar disorder.

In regard to the period of onset of the main ADRs, somnolence occurred in the first 7 days in 25 of 55 patients (45.45%), in 8–14 days in 11 (20.00%), in 15–28 days also in 11 (20.00%), in 29–56 in 4 (7.27%), in 57–84 in 3 (5.45%), and after 85 days or more in 1 patient (1.82%). Akathisia occurred in the first 7 days in 3 of 11 patients (27.27%), in 8–14 days in 3 (27.27%), in 15–28 in 2 (18.18%), 29–56 in 2 (18.18%), and 57–84 in 1 patient (9.09%). Dizziness occurred in the first 7 days in 5 of 10 patients (50.00%), in 8–14 days in 4 (40.00%), and in 15–28 days in 1 patient (10.00%). Lastly, somnolence—the most common ADR— recovered or improved after 1–7 days in 16 of 55 patients (29.09%), 8–14 days in 4 (7.27%), 15–28 in 13 (23.64%), 29–56 in 11 (20.00%), 57–84 in 8 (14.55%), and 85 days or more in 3 patients (5.45%).

#### 3.4.2 Factors influencing the occurrence of adverse drug reactions

The analysis of the patient characteristics increasing the risk of ADR occurrence revealed that “longer time since the onset of the first episode” (*p* = 0.011) and “presence of complications” (*p* < 0.001) were statistically significant risk factors (Table 3).

**TABLE 3:** Proportion of adverse drug reactions related to patient background factors.

Factor			Number of patients (%)	Number of patients who experienced adverse drug reaction (%)	Hypothesis testing

Safety analysis set patients		345	111 (32.17)	–
Sex	Male		128 (37.1)	40 (31.25)	(F) *p* = 0.812
	Female		217 (62.9)	71 (32.72)	
Age	<15 years		0 (0.0)	0 –	–
	15–19 years		8 (2.3)	1 (12.50)	
	20–29 years		73 (21.2)	23 (31.51)	
	30–39 years		75 (21.7)	30 (40.00)	
	40–49 years		80 (23.2)	23 (28.75)	
	50–59 years		64 (18.6)	24 (37.50)	
	60–64 years		14 (4.1)	2 (14.29)	
	≥65 years		31 (9.0)	8 (25.81)	
	Unknown		0 (0.0)	0 –	
Diagnosis	Bipolar I disorder		100 (29.0)	27 (27.00)	(F) *p* = 0.206
	Bipolar II disorder		245 (71.0)	84 (34.29)	
	Unknown		0 (0.0)	0 –	
BMI	<18.5 kg/m^2^		10 (2.9)	4 (40.00)	(*χ*) *p* = 0.958
	18.5 kg/m^2^–24 kg/m^2^		41 (11.9)	16 (39.02)	
	≥25 kg/m^2^		17 (4.9)	6 (35.29)	
	Unknown		277 (80.3)	85 (30.69)	
Care status at baseline	Inpatient		39 (11.3)	12 (30.77)	(F) *p* = 1.000
	Outpatient		306 (88.7)	99 (32.35)	
	Unknown		0 (0.0)	0 –	
Number of episodes in the past 12 months	<4		214 (62.0)	66 (30.84)	–
	≥4		9 (2.6)	4 (44.44)	
	Unknown		122 (35.4)	41 (33.61)	
	0		15 (4.3)	4 (26.67)	(F) *p* = 0.781
	≥1		208 (60.3)	66 (31.73)	
	Unknown		122 (35.4)	41 (33.61)	
Time since onset of first episode	<12 months		19 (5.5)	3 (15.79)	(C) *p* = 0.011
	12–23 months		18 (5.2)	3 (16.67)	
	24–35 months		13 (3.8)	3 (23.08)	
	36–59 months		25 (7.2)	7 (28.00)	
	60–119 months		55 (15.9)	19 (34.55)	
	≥120 months		109 (31.6)	41 (37.61)	
	Unknown		106 (30.7)	35 (33.02)	
Time since onset of current depressive episode	<3 months		169 (49.0)	55 (32.54)	(C) *p* = 0.501
	3–5 months		45 (13.0)	12 (26.67)	
	6–8 months		19 (5.5)	5 (26.32)	
	≥9 months		56(16.2)	22 (39.29)	
	Unknown		56 (16.2)	17 (30.36)	
Hepatic function at baseline	Healthy		71 (20.6)	22 (30.99)	–
	Impaired		8 (2.3)	0 (0.00)	
	Impairment grade	Mild	7 (2.0)	0 (0.00)	–
		Moderate	1 (0.3)	0 (0.00)	
		Severe	0 (0.0)	0 –	
	Unknown		266 (77.1)	89 (33.46)	
Renal function at baseline	Healthy		67 (19.4)	20 (29.85)	–
	Impaired		8 (2.3)	1 (12.50)	
	Impairment grade	Mild	8 (2.3)	1 (12.50)	–
		Moderate	0 (0.0)	0 –	
		Severe	0 (0.0)	0 –	
	Unknown		270 (78.3)	90 (33.33)	
Complication	Absent		193 (55.9)	40 (20.73)	(F) *p* < 0.001
	Present		140 (40.6)	68 (48.57)	
	Unknown		12 (3.5)	3 (25.00)	
Medical history	Absent		286 (82.9)	88 (30.77)	(F) *p* = 0.190
	Present		36 (10.4)	15 (41.67)	
	Unknown		23 (6.7)	8 (34.78)	

*Note*: (*χ*) chi–squared test, (F) Fisher's exact test, (C) Cochran‐Armitage trend test.

The ratio of ADR occurrence in relation to the diagnosis was 27/100 patients (27.00%) with bipolar I disorder and 84/245 patients (34.29%) with bipolar II disorder. As for a history of prior pharmacotherapy (antipsychotics or mood stabilizers), 24/67 patients (35.82%) with prior antipsychotic treatment experienced ADRs, compared with 87/278 (31.29%) with no previous antipsychotics; in 56/168 (33.33%) with previous mood stabilizer use, and in 55/177 (31.07%) with no mood stabilizer history.

Moreover, ADR occurred in 19/60 patients (31.67%) with concomitant use of antipsychotics and in 92/285 patients (32.28%) not using these drugs. The main ADRs experienced by patients taking antipsychotics were somnolence in 7/60 (11.67%) and constipation in 3/60 (5.00%), whereas in patients not using them, somnolence occurred in 48/285 (16.84%), and akathisia and dizziness in 10/285 each (3.51%). ADRs occurred in 55/177 patients taking mood stabilizers (31.07%) and 56/168 patients (33.33%) not using them. The most common ADRs in patients using mood stabilizers were somnolence in 30/177 (16.95%) and akathisia and dizziness in 5/177 (2.82%) each; 25/168 patients (14.88%) not using mood stabilizers incurred somnolence, 6/168 (3.57%) akathisia, and 5/168 each (2.98%) experienced dizziness and weight increase.

### 3.5 Efficacy

#### 3.5.1 Changes in MADRS score

**FIGURE 2:**
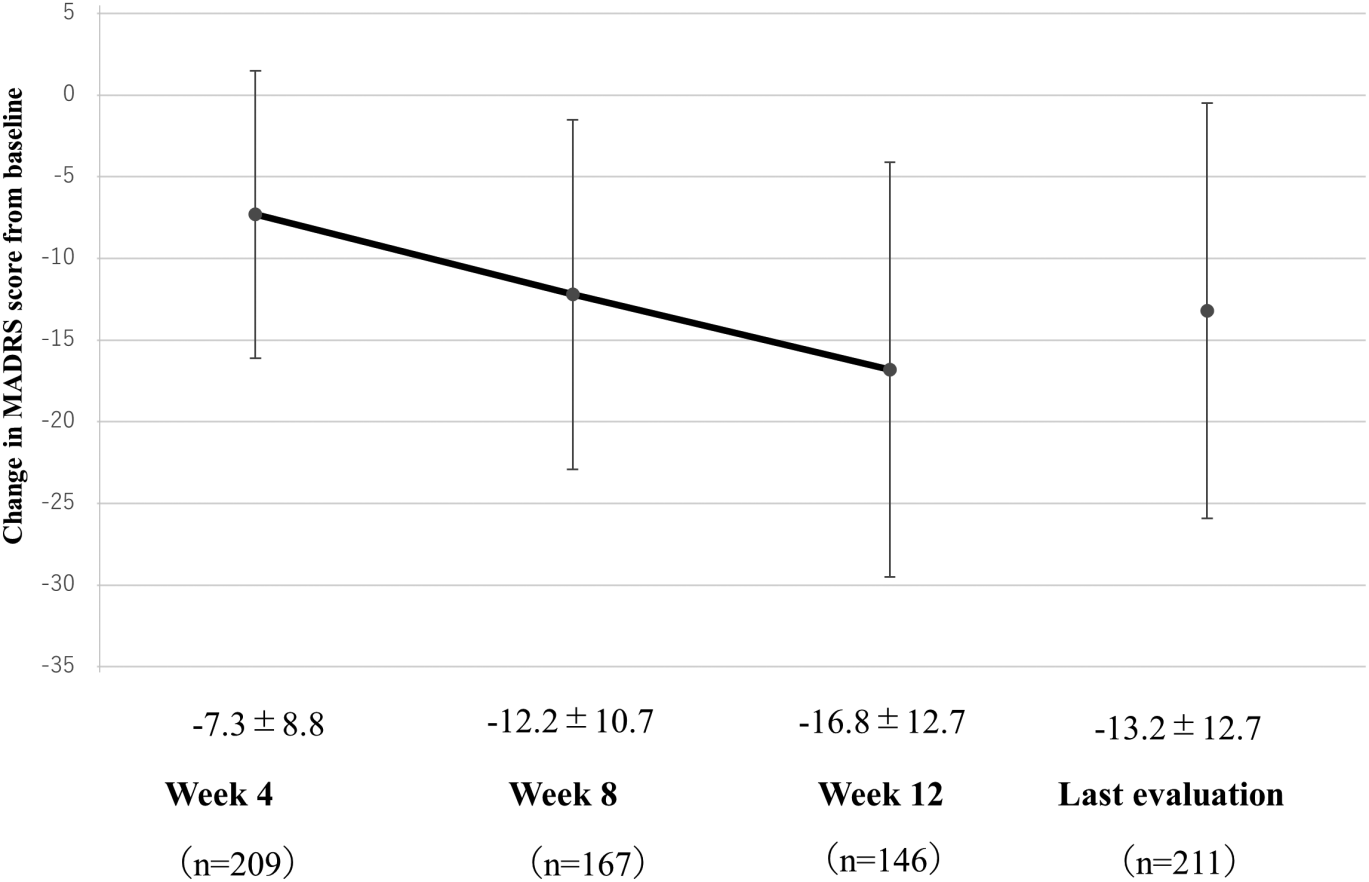
Differences in MADRS total score changes from baseline to last evaluation. The score on the Montgomery‐Åsberg Depression Rating Scale (MADRS) was assessed at baseline, and changes from the baseline were evaluated at Week 4, 8, 12, and last evaluation. The values are expressed as mean ± standard deviation.

**FIGURE 3:**
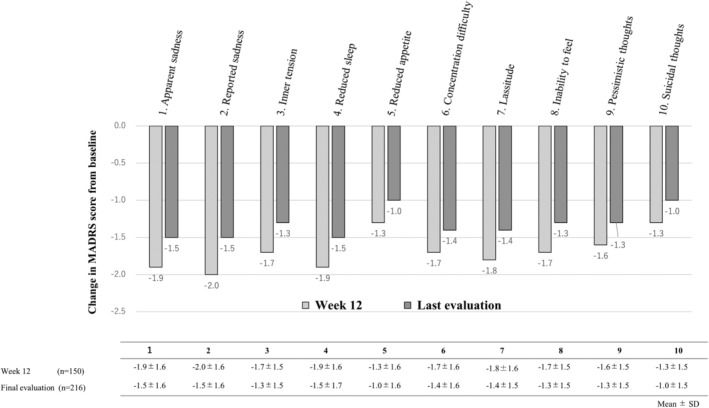
Changes in score from baseline for each item of the MADRS (mean). Due to large SD values, means are presented in the graph, while SD values are displayed in the table. The Montgomery‐Åsberg Depression Rating Scale (MADRS) comprises 10 items. This figure illustrates the average change from baseline at Week 12–the last evaluation for each of the 10 items.

The changes in the MADRS total score from baseline, variations at each time point, and change from baseline in each MADRS item are shown in Figure S1 and Figures 2 and 3, respectively.

The mean baseline score was 28.8 ± 11.1; after 4, 8, and 12 weeks and at the last evaluation, it was 21.5 ± 10.1, 17.1 ± 9.1, 13.0 ± 8.6, and 15.3 ± 9.8 points, respectively. Thus, the MADRS total score was lower at all time‐points than at baseline, with mean changes from baseline at the same timepoints of −7.3 ± 8.8, −12.2 ± 10.7, −16.8 ± 12.7, and − 13.2 ± 12.7 points, respectively (Figure 2). Furthermore, the mean change from baseline was negative for each MADRS item, and the score for all MADRS items was lower than at baseline both after 12 weeks and at the last evaluation (Figure 3).

#### 3.5.2 Factors possibly influencing the efficacy

##### Maximum daily dose

**FIGURE 4:**
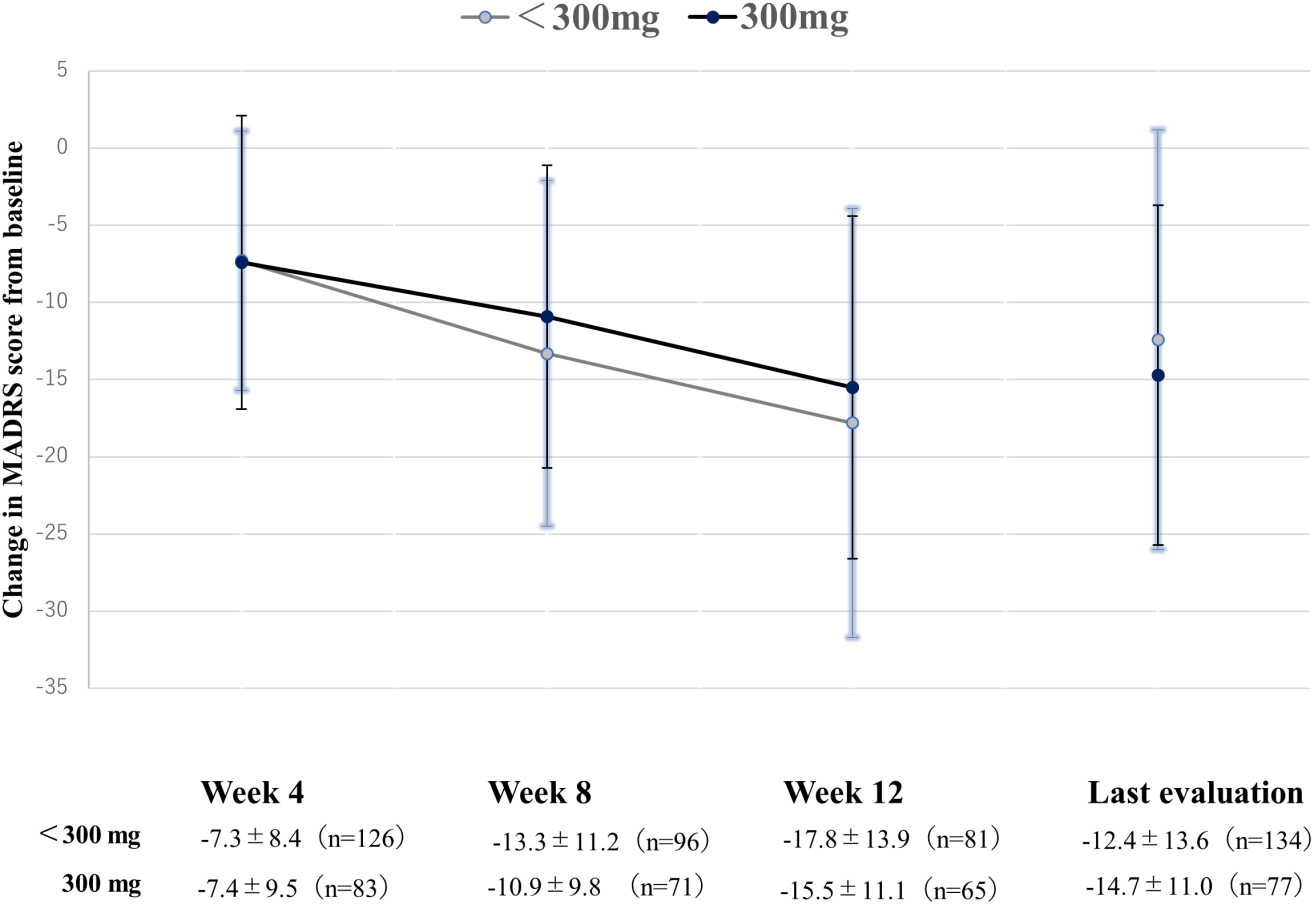
Differences in score change from the baseline MADRS total score in relation to the maximum daily dose of the study drug. The maximum daily dose approved in Japan for quetiapine extended‐release tablets is 300 mg. The score on the Montgomery‐Åsberg Depression Rating Scale (MADRS) was calculated at baseline, and changes from the baseline were evaluated at Week 4, 8, 12, and at last evaluation. The values are expressed as mean ± standard deviation.

Figure 4 shows the differences in mean MADRAS total score changes from the baseline according to the maximum daily dose.

The baseline MADRS total score was 28.6 ± 11.5 in the group of patients with the maximum daily dose of the study drug <300 mg, and 29.0 ± 10.2 in those with 300 mg; the change in score from baseline at the last evaluation for these groups was −12.4 ± 13.6 and −14.7 ± 11.0 points, respectively.

##### Diagnosis

**FIGURE 5:**
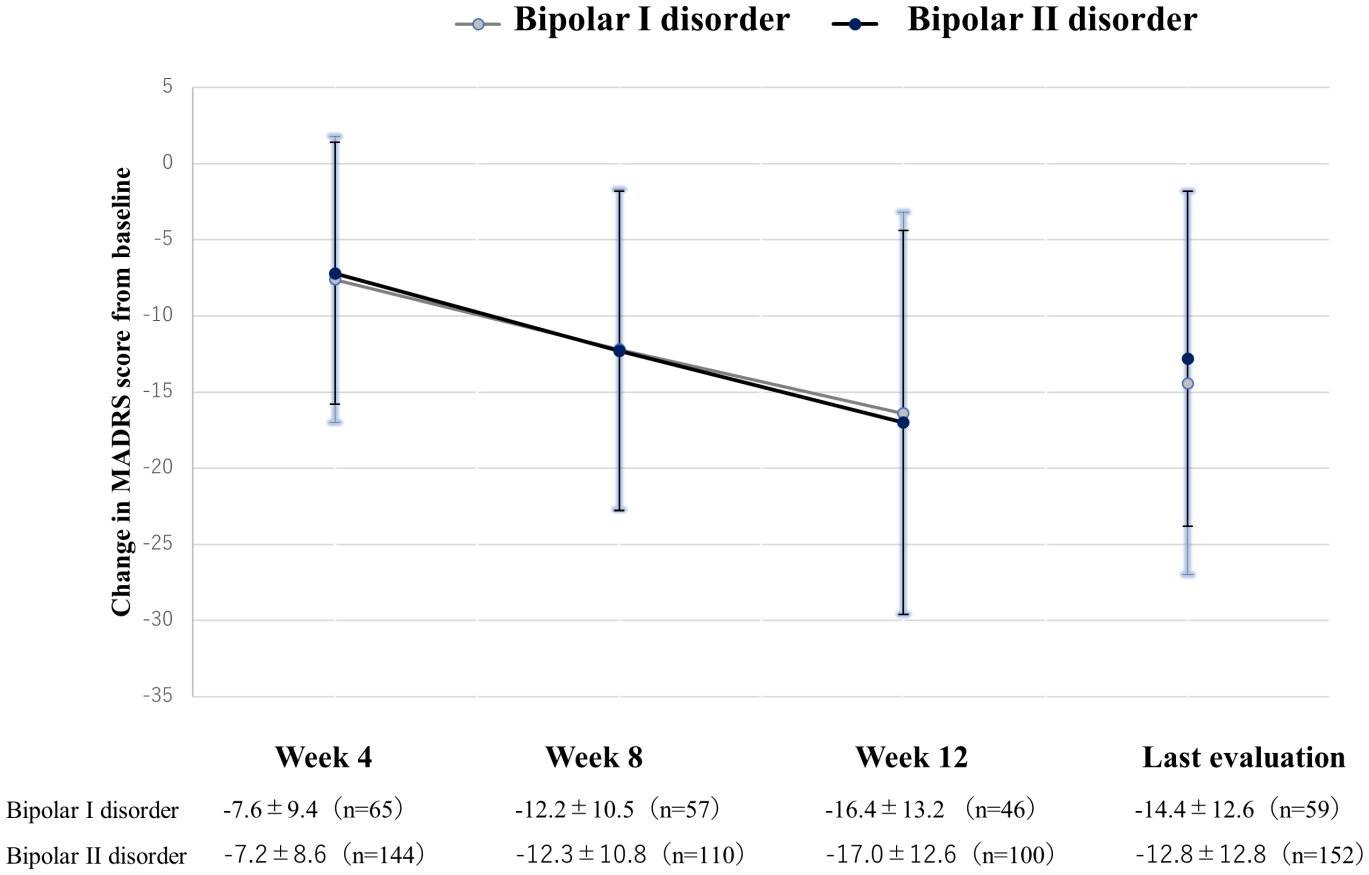
Differences in score change from the baseline MADRS total score in relation to the diagnosis. The score on the Montgomery‐Åsberg Depression Rating Scale (MADRS) was calculated at baseline, and changes from the baseline were evaluated at Week 4, 8, 12, and at last evaluation. The values are expressed as mean ± standard deviation.

Figure 5 shows the differences in mean MADRS total score changes from baseline based on the diagnosis (bipolar I or II disorder).

The baseline MADRS total score was 29.06 ± 11.8 in patients with bipolar I disorder and 28.6 ± 10.8 in those with bipolar II disorder; the change in score from baseline at the last evaluation was −14.4 ± 12.6 and −12.8 ± 12.8 points for these groups, respectively.

##### Prior pharmacotherapy (antipsychotics or mood stabilizers)

The baseline MADRS total score was 24.4 ± 9.2 and 29.9 ± 11.2 in patients who received prior pharmacotherapy with antipsychotics or not, respectively; the change in score from baseline at the last evaluation for these groups was −10.1 ± 9.8 and −14.1 ± 13.3 points, accordingly.

The baseline MADRS total score was 26.7 ± 9.2 and 31.0 ± 12.4 in patients with and without prior pharmacotherapy with mood stabilizers, and the change from baseline at the last evaluation was −11.7 ± 11.2 and −14.8 ± 14.1 points, respectively.

Figure S2 shows the differences in mean MADRS total score change from baseline based on the use of pharmacotherapy (antipsychotics or mood stabilizers) or not prior to use of the study drug.

##### Concomitant drugs (antipsychotics or mood stabilizers)

The baseline MADRS total score was 24.0 ± 9.1 and 29.9 ± 11.2 points in patients taking concomitant antipsychotics or not, and the change from baseline at the last evaluation was −9.8 ± 10.8 and −14.1 ± 13.0 points for these groups, respectively. The total score at baseline was 27.3 ± 9.5 and 30.5 ± 12.4 points in patients taking concomitant mood stabilizers or not, respectively, and the corresponding change in score at the last evaluation was −12.4 ± 11.5 and −14.3 ± 14.1 points.

Figure S3 shows the differences in mean change from baseline MADRS total score based on the use or not of concomitant drugs (antipsychotics or mood stabilizers).

##### Other influential factors

The number of older patients, patients with renal dysfunction, and those with liver dysfunction was very small. The change from baseline at the last evaluation by the MADRS total score in older patients, patients with renal dysfunction, and those with hepatic dysfunction were − 17.5 ± 17.3 (*n* = 17), −14.5 ± 11.9 (*n* = 4), and − 14.7 ± 17.9 (*n* = 6), respectively.

### 3.6 Response and remission proportions

The proportions of patients demonstrating a positive response at 4, 8, and 12 weeks and at the last evaluation were 33/209 (15.8%), 66/167 (39.5%), 84/146 (57.5%), and 98/211 (46.4%), respectively. Moreover, 16/242 patients (6.6%) were in remission at baseline, whereas the remission proportions at 4, 8, and 12 weeks and at the last evaluation were 33/213 patients (15.5%), 56/172 (32.6%), 80/150 (53.3%), and 97/216 (44.9%), respectively.

## 4 DISCUSSION

The overall proportion of occurrence of ADRs in the present study was 111/345 patients (32.17%), lower than the proportion of 287/341 patients (84.16%) reported in a clinical trial^11^ at the time of drug approval. The most common ADRs were somnolence, akathisia, dizziness, weight increase, thirst, hypersomnia, constipation, and nausea; these ADRs were also observed in the clinical trial mentioned above, and all were mild. The only serious ADR was one patient of suicidal ideation. Somnolence, the most frequent ADR, occurred in the first 7 days for half of the patients who experienced it, and mostly recovered within 28 days.

Safety specifications that should be specifically evaluated at the time of approval include “hyperglycemia, diabetic ketoacidosis, or diabetic coma,” “hypoglycemia,” “neuroleptic malignant syndrome,” “rhabdomyolysis,” “seizure,” “agranulocytosis or leukopenia,” “hepatic impairment or jaundice,” “paralytic ileus,” “tardive dyskinesia,” “pulmonary embolism or deep vein thrombosis,” “withdrawal syndrome,” “lipid metabolism disorder,” “suicidal ideation or suicidal behavior,” “hostility or aggression,” “urinary retention,” “long QT syndrome,” and “pancreatitis.” In our study, a severe ADR of “suicidal ideation or suicidal behavior” occurred in one patient; a milder “hepatic impairment or jaundice” was noted in three patients, and “hyperglycemia, diabetic ketoacidosis, or diabetic coma,” and “lipid metabolism disorder” were experienced by one patient each.

The analysis of risk factors for ADR occurrence showed that “longer time since the onset of the first episode” and “presence of complications” were significant factors, whereas “diagnosis,” “presence or absence of prior pharmacotherapy,” and “presence or absence of concomitant drugs or therapies” showed no statistically significant difference. These results suggest that the proportion of occurrence of ADRs is higher after a longer time since the onset of the patient's first episode and in the presence of complications. However, patients with complications are more likely to be in poor conditions than otherwise healthy patients, and this underlying situation may be a more relevant factor in the higher proportion of ADR occurrence in the former group.

Our study found that mood switches were not observed. A recent network meta‐analysis demonstrated that quetiapine was associated with a lower incidence of manic switching compared to a placebo.^6^ This result is crucial to manage the individuals with bipolar disorder in the clinical practice.

The typical dosage of quetiapine in adults starts at 50 mg and is increased to 150 mg after an interval of at least 2 days. Then, after an additional interval of at least 2 days, the dosage can be further increased to the recommended 300 mg. In all patients, the drug is administered orally once daily before bedtime and at least 2 h after eating. This dosing schedule was determined based on a previous study that examined the plasma drug concentration in 24 healthy non‐elderly men receiving a single 50 mg dose of the study drug, either fasting or after eating a low‐ or high‐fat meal; the concentration was generally higher after eating than after fasting.^12^ The gradual increase to a recommended dose of 300 mg/day was also established based on a phase‐III non‐Japanese trial reporting no clear advantage from increasing the dose beyond 300 mg/day.^13^ In addition, a network meta‐analysis found no significant difference in efficacy between a maximum dose of 600 mg/day (quetiapine immediate release group) and 300 mg/day (extended release); in addition, the 600 mg/day dose was poorly tolerated.^14^

In the present study, more than 90% of patients started the treatment at a dose of 50 mg/day; approximately half of them increased to 150 mg/day, and a quarter to 300 mg/day. None of the patients received a dose exceeding the recommended 300 mg/day. Furthermore, the Japanese guidelines regarding concomitant therapies only weakly recommend the combination of second‐generation antipsychotics and mood stabilizers or the combination of multiple mood stabilizers for patients with bipolar depressive episodes (overall evidence level: 2C);^8^ less than 60% of patients were using these drug combinations. In total, 177 patients were using concomitant mood stabilizers and 60 concomitant antipsychotics, and the analysis of the study drug's efficacy in relation to concomitant use or not of each drug type revealed similar decreasing changes in the MADRS total score from baseline, regardless of the use of concomitant drugs, and no differences in the occurrence of ADRs.

These results demonstrate that the mean MADRS total score decreased from baseline in the efficacy analysis group, irrespective of maximum daily dose, diagnosis, and whether prior or concomitant pharmacotherapy was used.

The MADRS total score in the present study showed a mean change of −13.2 ± 12.7 between baseline (28.8 ± 11.1) and last evaluation. This improvement is comparable to the decrease seen in the Japanese phase‐II/III clinical trials (CL‐0021),^11^ where the MADRS total score for the 300 mg/day group decreased by 12.6 ± 11.4 points between baseline (30.9 ± 6.9) and the last evaluation of the treatment phase I (week 8). Furthermore, the response and remission proportions were 79/179 patients (44.1%) and 68/179 patients (38.0%), respectively, at the last evaluation of the treatment phase I for the 300 mg/day group in CL‐0021, compared with 98/211 patients (46.4%) and 97/216 patients (44.9%) at the last evaluation in the present study. A direct comparison between these results is not feasible due to differing patient attributes; however, these findings seem to suggest that the improvements in MADRS total score from baseline are similar in clinical trials and real‐world settings.

This study was observational, and some limitations must be considered. The study was non‐interventional and with no control group, based on reports provided by physicians, thus inconsistencies in the judgments by each doctor are possible. It was based on a 12‐week follow‐up period and did not investigate the long‐term use. Also, the inclusion and exclusion criteria were not rigorous, and as a result the patient characteristics were diverse. Concomitant drugs were not restricted, and the impact of such drugs could not be completely eliminated. Last, the reports were recorded under real clinical conditions; therefore, clinical test results were not available for all patients.

In conclusion, this post‐marketing surveillance of the real‐world safety and efficacy of Bipresso® extended‐release tablets suggests that this drug is effective in patients experiencing depressive symptoms of bipolar disorder, and no new safety concerns or risks concerning ADRs emerged in the period considered.

## AUTHOR CONTRIBUTIONS

Astellas Pharma Inc. (Tokyo, Japan) designed the original study, developed the protocol, and performed the data analysis of Bipresso® extended‐release tablets. Mr. Irie, Mr. Aikawa, and Dr. Kishi wrote the manuscript. Dr. Iwata supervised the review. All authors were involved in the decision to submit this article for publication, contributed to the interpretation, reviewed the manuscript, and approved the final version.

## FUNDING INFORMATION

This study was funded by KYOWA Pharmaceutical Industry Co., Ltd.

## CONFLICT OF INTEREST STATEMENT

Dr. Kishi has received speaker's honoraria from Sumitomo, Eisai, Janssen, Otsuka, Meiji, MSD, Viatris, and Takeda and research grants from Eisai, the Japanese Ministry of Health, Labour and Welfare (21GC1018), Grant‐in‐Aid for Scientific Research (C) (19 K08082), and the Japan Agency for Medical Research and Development (JP22dk0307107 and JP22wm0525024). Dr. Iwata received speaker's honoraria from Sumitomo, Eisai, Janssen, Otsuka, Meiji, Shionogi, Takeda, Yoshitomiyakuhin, and Viatris, and research grants from Eisai, Takeda, Sumitomo, and Otsuka. Irie and Aikawa are employees of KYOWA Pharmaceutical Industry Co., Ltd.

## ETHICS STATEMENT

The study was conducted in accordance with the Good Post‐marketing Study Practice in Japan.

Informed consent: Not applicable.

## CLINICAL TRIAL REGISTRATION

Not applicable.

## PATIENT CONSENT STATEMENT

Studies conducted in accordance with the Good Post‐marketing Study Practice in Japan do not require informed consent.

## Supporting information


Figure S1.

Figure S2.

Figure S3.

## Data Availability

The data for this surveillance are not available in a public repository because KYOWA Pharmaceutical Industry Co., Ltd. takes suitable measures to protect personal information and the sponsor's intellectual property. The nature of the information protected will be tailored to the specific request.
