# Supplementary figures and images for: Post‐marketing surveillance of quetiapine fumarate extended‐release tablets in patients with bipolar depression
Source: Neuropsychopharmacol Rep. 2024 Apr 30;44(2):424–36. doi: 10.1002/npr2.12441 (PMC11144599; doi:10.1002/npr2.12441)

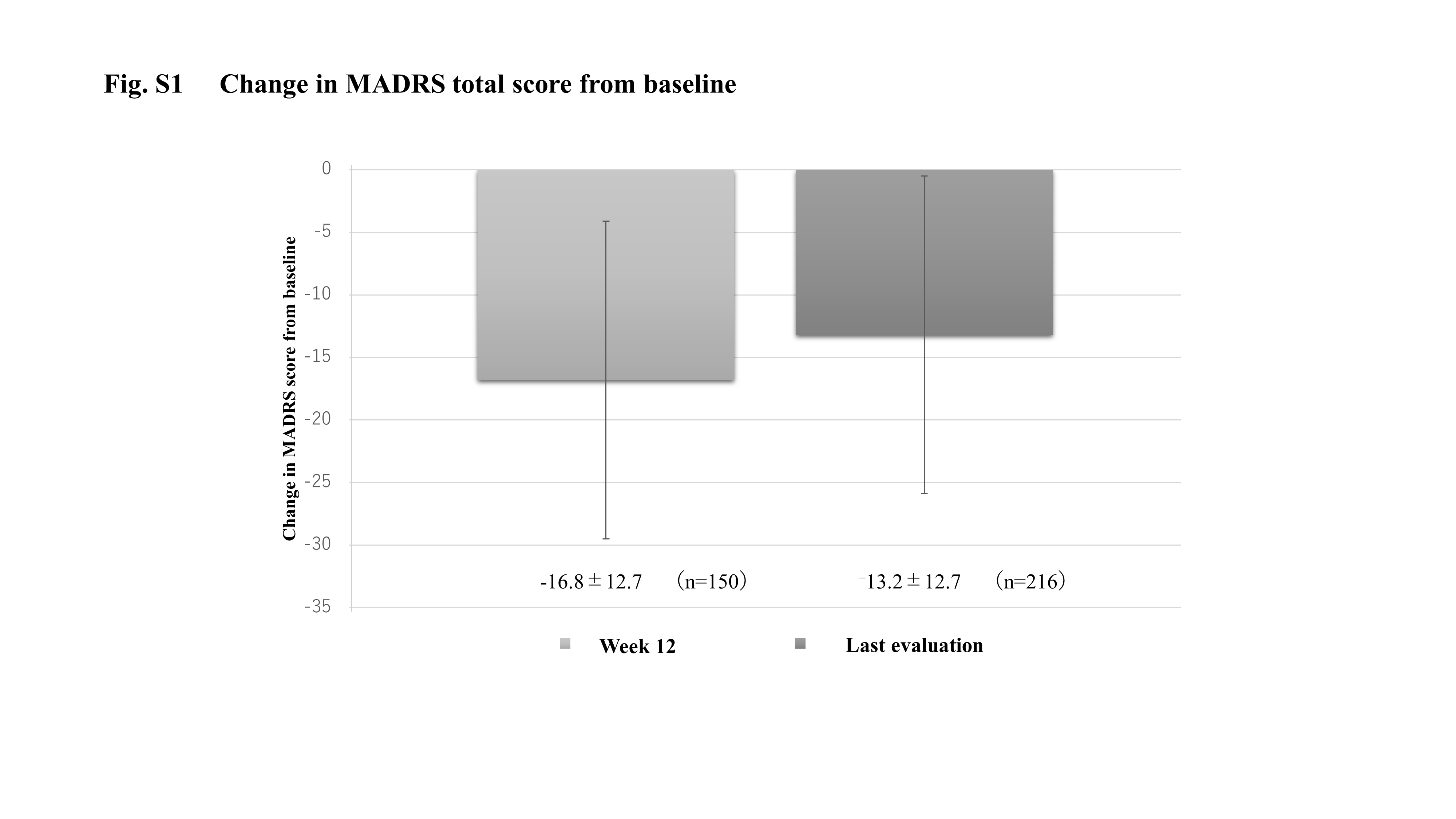

Supplement: Supplementary file 1 — Figure S1. [file NPR2-44-424-s003.tif]

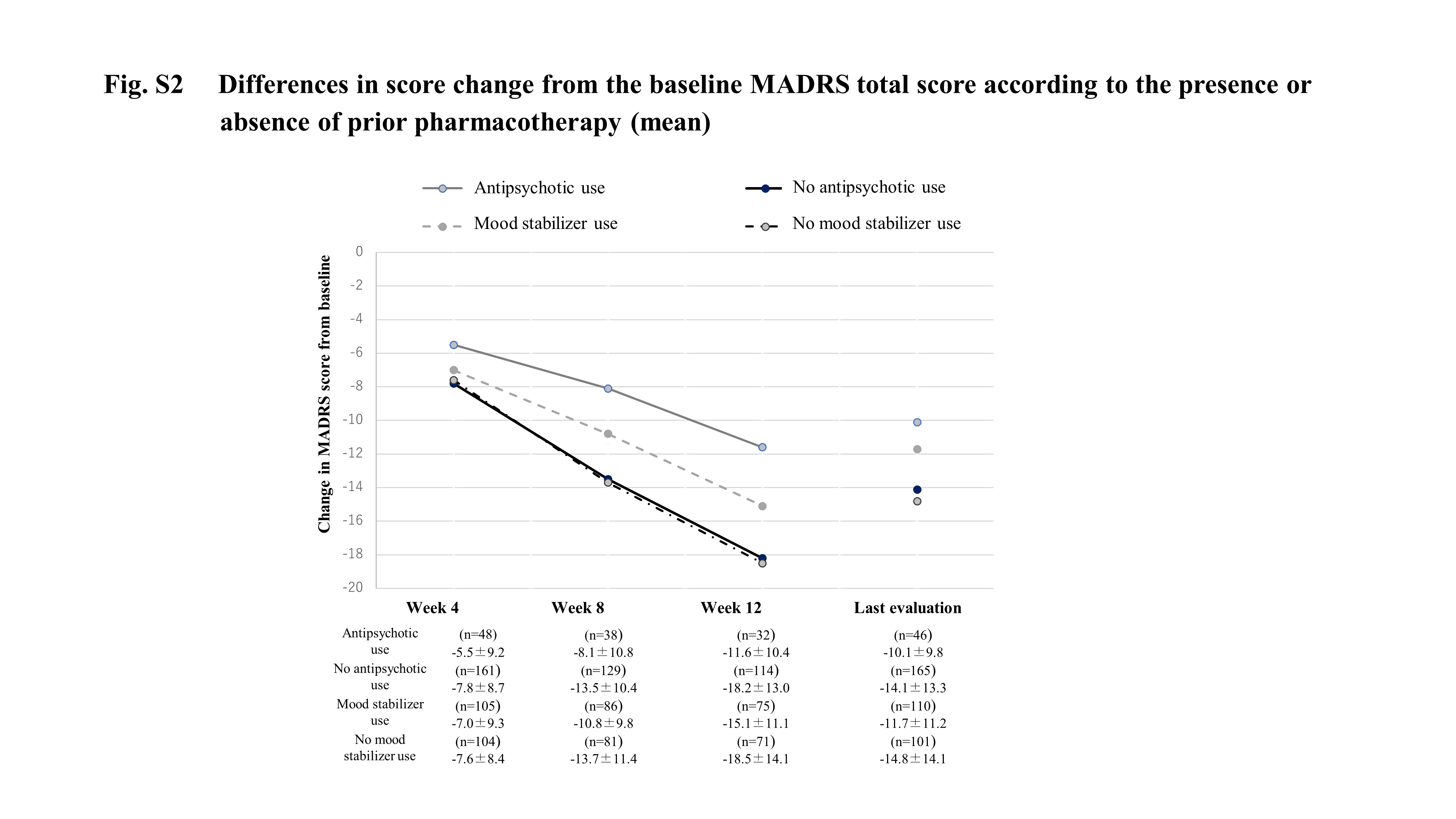

Supplement: Supplementary file 2 — Figure S2. [file NPR2-44-424-s001.tif]

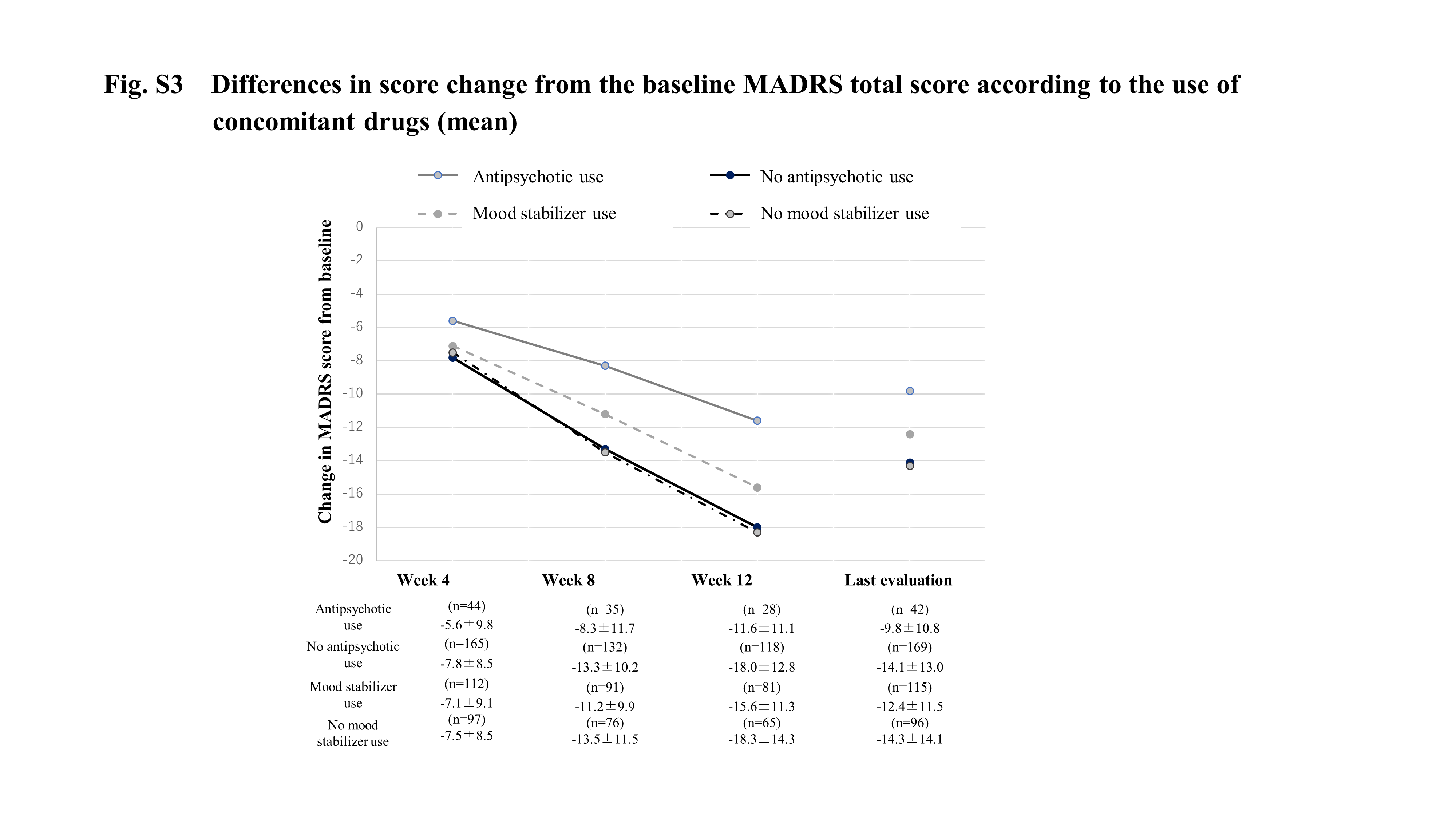

Supplement: Supplementary file 3 — Figure S3. [file NPR2-44-424-s002.tif]
